# A Longitudinal Pilot Study of Behavioral Abnormalities in Children with Autism

**DOI:** 10.26502/jppd.2572-519X0022

**Published:** 2017-07-12

**Authors:** Robin A. Libove, Thomas W. Frazier, Ruth O’Hara, Jennifer M. Phillips, Booil Jo, Antonio Y. Hardan

**Affiliations:** 1 Department of Psychiatry and Behavioral Science, Stanford University School of Medicine, 401 Quarry Road, Stanford, CA, 94305, USA; 2 Center for Autism, Pediatric Institute and Cleveland Clinic Lerner College of Medicine, 2801 Martin Luther King Jr. Drive, Cleveland, OH, 44104, USA

**Keywords:** Child Behavior Checklist, Social, Externalizing, Aggressive, Oppositional defiant, Development

## Abstract

This longitudinal investigation examined the development of emotional and behavioral functioning in school-age children with autism. The Child Behavior Checklist was obtained at baseline and after an average interval of 28.5 months from 13 boys with autism and 14 age- and gender-matched controls between the ages of 7 and 12 years at baseline. Children with autism demonstrated clinically significant elevations in several domains including Social, Thought, and Attention Problems. Children with autism exhibited significant improvements over time in Total, Externalizing, Social, and Oppositional Defiant Problems and Aggressive Behavior, while there were no changes over time in the controls. These findings suggest that children with autism may demonstrate improvements over time in some clinical domains such as social and behavioral functioning.

## 1. Introduction

Autism is a neurodevelopmental disorder characterized by the core symptoms of social/communication impairments, and repetitive behaviors [1]. Comorbid psychopathology, such as anxiety, attention- deficit/hyperactivity, and oppositional defiant disorders, is commonly present in individuals with this condition [2–6]. Autism is frequently described as a chronic condition with some individuals showing improvements in some core symptoms and associated behaviors over time [5, 7–9]. Previous studies suggest that many children with autism, while they continue to meet diagnostic criteria, can demonstrate improvements in social interaction, reduction in repetitive behaviors, as well as changes in maladaptive behaviors as they get older [7, 10]. Longitudinal studies have found that children’s autism symptoms often change as they mature and become adolescents and adult [8, 10, 11].

To date, however, a limited number of investigations have focused on the course of specific emotional and behavioral changes over time using longitudinal methodology. Further, previous studies have primarily used cross-sectional designs or have focused on development in adolescence or adulthood. In a study by Kanne at al. [12], which examined behavioral problems in a sample of children and adolescents aged 3 to 18 years with autism spectrum disorders, parents reported substantial comorbidity with affective (26%), anxiety (25%), attentional (25%), conduct (16%), oppositional (15%), and somatic problems (6%) as measured by the Child Behavior Checklist (CBCL). Moreover, a study by Simonoff et al. [13] found that 70% of a population based sample of children with autism ages ten to 14-years had at least one comorbid disorder and 41% had two comorbid disorders. The most frequent comorbid conditions in this sample included social anxiety, attention-deficit/hyperactivity disorder, and oppositional defiant disorder [13].

Examining the development of problem behaviors such as aggression, hyperactivity, and anxiety is critical for understanding the course of autism spectrum disorders. Behavioral characterization of development across the lifespan is essential to understanding this life-long condition and implementing appropriate interventions at a critical time of development to optimize outcome. The goal of the present pilot investigation was to examine the changes in emotional and behavioral functioning over a period of 2–3 years, in school-age children with autism compared to typically developing controls.

## 2. Methods

### 2.1 Participants

The sample included 13 boys with autism and 14 age- and gender-matched healthy control youth who were participants in a larger longitudinal neuroimaging study [14]. Parents completed the Child Behavior Checklist for ages 6–18 years [15] at baseline (Time 1) and follow-up (Time 2), with an average test-retest interval of 28.5 months. The Wechsler Intelligence Scale for Children (WISC-III) [16] was administered to measure cognitive functioning for all subjects. All subjects had a FSIQ > 70 and academic skills for controls were assessed using the Wide Range Achievement Test-Revised [17].

The diagnosis of autism was established through expert clinical evaluation and confirmed by two structured research diagnostic instruments including the Autism Diagnostic Interview-Revised (ADI-R) [18] and the Autism Diagnostic Observation Schedule (ADOS) [19]. Children with secondary autism such as those diagnosed with tuberous sclerosis and Fragile X were excluded.

Controls subjects were children recruited from the community through advertisements in areas socioeconomically comparable to those of the families of origin of subjects with autism. Control subjects were screened by in person interviews, questionnaires, telephone interviews, and observation during psychometric testing. Individuals with a family history of any neuropsychiatric disorder, such as autism, learning disability, affective disorders, and schizophrenia, were not included. Potential subjects with a history of birth asphyxia, head injury, or a seizure disorder were also excluded. Informed consent was obtained from all parents and children participants provided assent if they were capable. The institutional review board approved the methodology of this study.

### 2.2 Measures

The CBCL/6–18 is a parent-report measure used to assess maladaptive behaviors in school-age children between the ages of 6 and 18 years. The CBCL/6–18 has 118 items that describe current emotional and behavioral functioning and test-retest reliability is high (>.80) for most scales [15]. Parents are asked to assess the frequency of their child’s specific behaviors using a 3-point Likert scale (0 = not true; 1 = somewhat or sometimes true; and 2 = very true or often true). The items provide total and T-scores for eight syndrome scales (Aggressive Behavior; Anxious/Depressed; Attention Problems; Rule-Breaking Behavior; Social Problems; Somatic Complaints; Thought Problems; and Withdrawn/Depressed), six DSM-oriented scales (Affective Problems; Anxiety Problems; Somatic Problems; Attention Deficit/Hyperactivity Problems; Oppositional Defiant Problems; and Conduct Problems), internalizing problems, externalizing problems, and total problems. A T-score of 70 or above on any of the scales is considered a clinically significant elevation, a T-score between 65 and 69 is in the borderline clinical range, and a T-score below 65 is in the normal range [15]. This study considered a T-score of 70 or above to be clinical significant and was included in our analyses. Socioeconomic status for all subjects was assessed using the Hollingshead method [20].

### 2.3 Data Analysis

Between-group differences on demographic data were assessed using Student’s T-tests. To examine change over time, we conducted a mixed model regression analysis separately for controls and children with autism, with CBCL mean score as the dependent variable. We ran separate mixed models for controls and subjects with autism due to the limited sample sizes, and minimal variance in the control group. Given the limited variance, for the controls, we were able to run a linear mixed model with random intercept only. For the autism group, we performed a linear mixed model with random intercept and random slope. Age was included in the mixed model analyses. Mixed models have the advantages of accounting for missing data across time points and explicitly models the effects of age rather than simply time of assessment. Multivariate analysis of covariance (MANCOVA) was then also preformed with group as independent variable, in order to control for confounding factors including receiving individual therapy and changes in medication. IQ was not used as a covariate in our analysis because previous studies [21–23], have indicated that there are associations between IQ and symptom severity; therefore including IQ as a covariate would remove the variance associated with autistic traits.

## 3. Results

The mean age of participants (autism and controls) was 10.6 ± 1.4 (Mean ± SD) years at baseline and 13.0 ± 1.7 years at follow-up. The autism group had an age range of 8–12 (mean: 11.0 ± 1.3) years at baseline and 9–16 (mean: 13.6 ± 1.7) years at follow-up. The controls had an age range of 7–12 (mean: 10.3 ± 1.4) years at baseline and 10–14 (mean: 12.4 ± 1.4) years at follow-up. No significant between-group differences (autism vs. control) were found on demographic data except, as expected, for the IQ measures including full scale (p= <.001); performance (p= <.001); and verbal IQ (p= .02). Of the children with autism included, 30.7% were receiving individual therapy and 23.0% had a change in medication during the time period between baseline and follow-up.

Children with autism demonstrated clinically significant elevations (i.e. T scores ≥ 70) in several behavioral domains on the CBCL at baseline, including Social Problems, Thought Problems, and Attention Problems. In addition, at baseline borderline elevations were noted in the autism group in the areas of Total Problem Behaviors, Anxious/Depressed behaviors, and Aggressive Behavior, as well as in the Composite domains for Anxiety Problems and Attention Deficit/Hyperactivity problems (see Table 1). However, there were statistically significant differences between the autism and control groups on all CBCL scales (ranges: F=6.32–86.76; p=<0.001–0.01). Among controls, no scores on the CBCL were clinically elevated.

The mixed model indicated that control subjects varied significantly with respect to their baseline scores on the CBCL (SE=14.86; Z=1.75; p<.04), but that this effect did not appear to vary according to age and scores were stable over time. However, subjects with autism had significant variation in their baseline performance (SE=449.01; Z=2.55; p<.005), and scores decreased significantly over time (SE=2.09; t=−2.56; p<.02). Specifically, eight children with autism showed significant improvement (decreasing scores), three had no change in behavior over time, while the performance of three children worsened. The MANCOVA controlling for confounding factors including receiving individual therapy (F=5.764; df=17; p=.020) and changes in medication (F=5.583; df=17; p=.033) revealed no significant main effects or interactions of these variables on changes over time on the CBCL scales.

Despite improvement in behavior over time, children with autism continued to demonstrate clinically significant levels of impairment in the area of Thought Problems and borderline elevations in Attention Problems, as well as in the Composite domains of Anxiety Disorders and ADHD. In contrast, no scores were clinically elevated in the control sample, either at baseline or follow-up.

## 4. Discussion

This pilot longitudinal investigation examined changes over time in emotional and behavioral functioning in a sample of high functioning school-age boys with autism. Consistent with previous cross-sectional studies, the participants with autism demonstrated social and behavioral deficits greater than those seen in their typical age-mates, as evidenced by clinical elevations on the CBCL in the areas of Social, Thought, and Attention Problems [13, 24]. Changes over time were also observed with significant within-subject changes on the total problems on CBCL scale in individuals with autism. Externalizing Problems, Social Problems, Aggressive Behavior, and Oppositional Defiant Problems appear to have the greatest decline. In contrast, and as expected, control children had normal levels of emotional and behavioral functioning at baseline, and exhibited no significant change over time.

Our findings suggest that over time children with autism demonstrate improvements in social and behavioral functioning, including reductions in social problems, as well as reductions in disruptive, aggressive, and maladaptive behaviors. It still remains unclear whether these improvements are related to therapeutic interventions, the course of the disorder, or both. However, when controlling for therapeutic interventions (individual therapy and/or changes in medications) over time no relationships were found. In contrast, the Anxious/Depressed domain in the autism group did not show a significant reduction over time, highlighting the potential clinical significance of these symptoms and the importance of treating these problems. Consistent with our findings, a cross-sectional investigation by Kuusikko and colleagues [25] revealed an increase in social anxiety and behavioral avoidance in high functioning children and adolescents with autism as they grew older and as measured by both parent and self-report. These observations highlight the importance of cognitive, behavioral, and pharmacological interventions in targeting comorbid anxiety symptoms observed in children with autism.

There were several methodological limitations in the present study. Specifically, our study was restricted to a small sample of boys in a relatively narrow age range and did not include children with intellectual disabilities. Not all participants in the study had CBCL data at both time-points. Girls were not included in this sample; therefore, the findings of this study cannot be generalized to all individuals with autism especially because the severity of maladaptive behaviors and the developmental trajectories may differ in females. Gender differences may be important to examine in future studies since there is conflicting evidence about the severity of maladaptive behaviors in females compared to males with AD [3, 8]. However, there is some evidence that high functioning females with autism have more social, attention, and thought problems when compared to males aged 5–20 years [26]. Furthermore, multiple data points would have allowed for a much better representation of the developmental trajectories of psychopathology and problem behaviors in children with autism.

Despite the limitations discussed above, the data presented here provide preliminary evidence supporting overall behavioral improvement over time, in some externalizing behaviors such as aggression in school-aged boys with autism. Future studies are needed to evaluate changes in behaviors in lower functioning individuals with IQ scores below 70 and across different age groups including early childhood, adolescences and adulthood. This would allow for better understanding developmental trajectories of problem behaviors across the lifespan. Lastly, parent-report measures used in conjunction with self-report measures and in-person interviews assessing behavioral symptoms would allow for a more comprehensive assessment of comorbid behavioral abnormalities.
